# The Hypoxic Tricuspid: A Flail Tricuspid Valve With Patent Foramen Ovale

**DOI:** 10.7759/cureus.41955

**Published:** 2023-07-16

**Authors:** Michael Ghaly, Joel Raja, Mannu Nayyar

**Affiliations:** 1 Internal Medicine, Baptist Memorial Hospital, Oxford, USA; 2 Cardiology, The University of Tennessee Health Science Center, Memphis, USA

**Keywords:** patent foramen ovale, tricuspid regurgitation, right-to-left shunt, right-sided heart failure, congestive heart faiulre, patent foramen ovale (pfo), flail tricuspid valve

## Abstract

Etiologies of tricuspid regurgitation are often explored in patients with symptoms of right-sided heart failure. Blunt chest trauma is the major cause of traumatic tricuspid valve regurgitation (TTVR), a secondary type of tricuspid regurgitation. It is a rare condition; however, it may lead to severe consequences if not treated in a timely manner. TTVR should be considered in a patient presenting with chest trauma. In this case, we report a case of a young male who presented after a motor vehicle accident with secondary tricuspid valve regurgitation due to blunt chest trauma as well as a patent foramen ovale.

## Introduction

Approximately 65-85% of adults have tricuspid valve regurgitation (TR), with about 1,600,000 patients who have moderate to severe TR [1]. TR is primarily diagnosed with an echocardiogram. Tricuspid regurgitation is usually divided into two categories, i.e., primary and secondary. Primary TR is caused by disease of the components of the tricuspid valve leaflets and chordae themselves, including rheumatic, congenital, endocarditis, and carcinoid heart disease. Secondary TR, which accounts for 90% of the cases, is from the dilation of the right ventricle leading to leaflet tethering and tricuspid annulus dilation [2]. A rare minority of patients having secondary TR may have flail TR. Flail TR is most commonly seen in patients with blunt chest trauma. Common signs experienced by patients can be subtle and nonspecific, such as hepatosplenomegaly, ascites, peripheral edema, and fatigue [3]. In 1829, the first case of traumatic TR was reported by Williams [2]. Although it may be underdiagnosed, traumatic TR occurs in about 0.02% of traumatic injuries [4].

Pulmonary hypertension may lead to secondary tricuspid regurgitation. Severe tricuspid regurgitation may be seen in patients with pulmonary hypertension as increased pressure and volume on the right ventricle will lead to dilation and eventually failure [5]. As the right ventricle becomes enlarged, the tricuspid valve may stretch and become incompetent [5]. Common conditions leading to pulmonary hypertension include left-sided heart failure, pulmonary disease, left-to-right shunt, including atrial and ventricular septal defects, Eisenmenger syndrome, as well as pulmonic valve stenosis.

## Case presentation

We present a case of a 38-year-old male with no known past medical or surgical history. He presented after being involved in a motor vehicle accident. On the initial presentation, he was profoundly hypotensive. The patient was arousable and responsive. Intravenous fluids and blood product resuscitation were started with stabilization of hemodynamics. A pelvic X-ray revealed large pubic symphysis diastasis. Orthopedic surgery was consulted and they placed a pelvic binder with a good reduction in the diastasis. Foley placement yielded frank hematuria. Full trauma scans were performed, which demonstrated extraperitoneal bladder rupture. Pelvic blush was also identified on CT.

Interventional radiology was consulted for pelvic angiography. This study revealed a left internal pudendal artery injury, which was embolized. The patient also underwent empiric right internal iliac embolization. Post-procedure, the patient became acutely hypoxic requiring bagging and maximum ventilator settings due to difficulty with oxygenation. Bilateral chest tubes were placed. A chest X-ray was unremarkable. The patient was placed on bilevel ventilation and nitric oxide was started.

**Figure 1:**
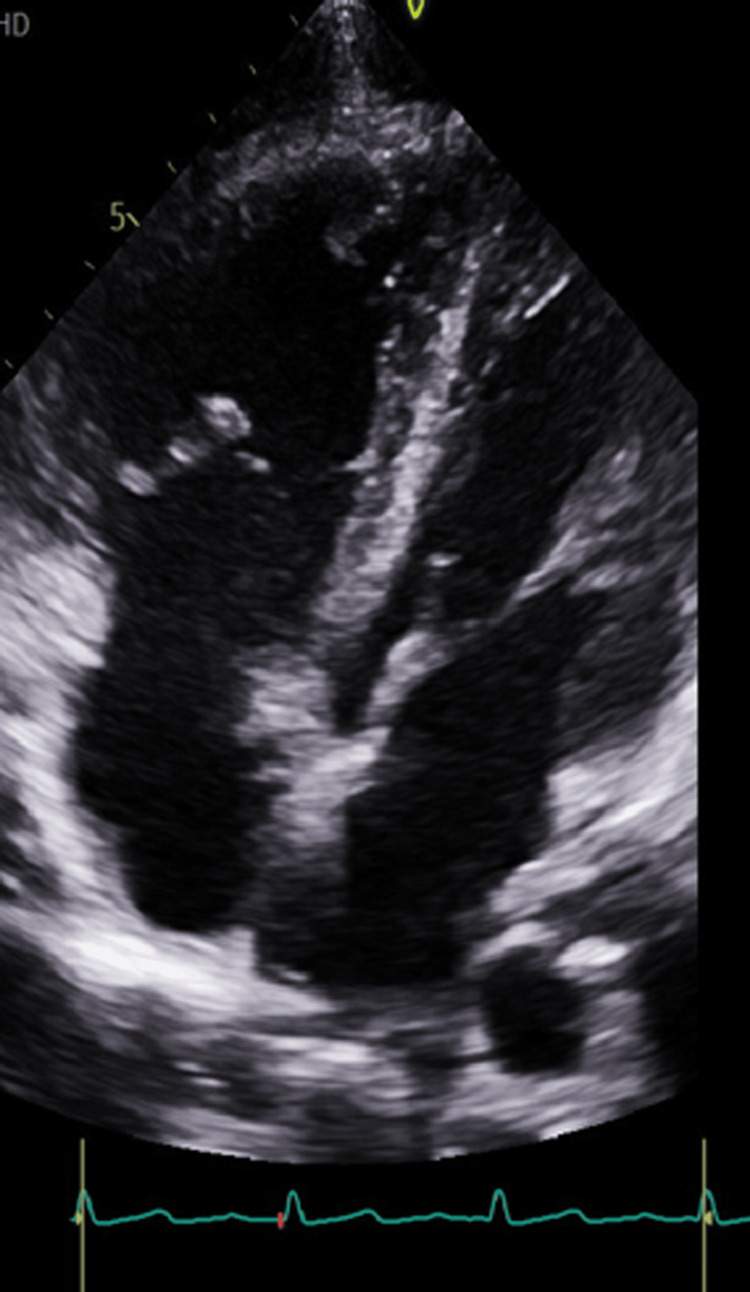
Transthoracic echocardiogram demonstrating flail anterior tricuspid leaflet with avulsed papillary muscle

**Figure 2:**
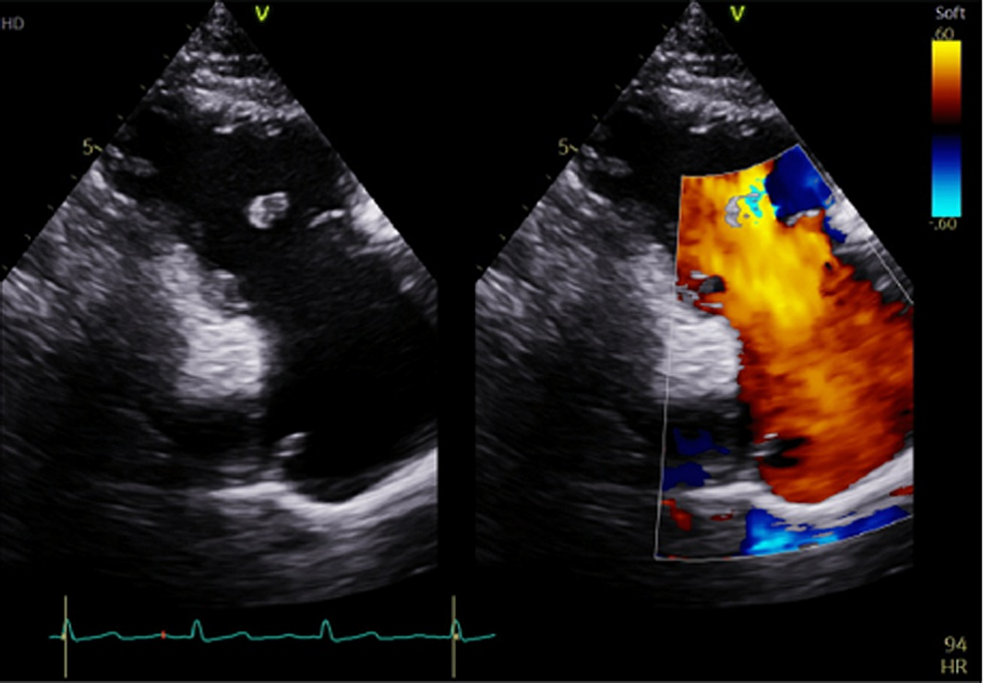
Flail anterior leaflet with torrential tricuspid regurgitation on color Doppler

**Figure 3:**
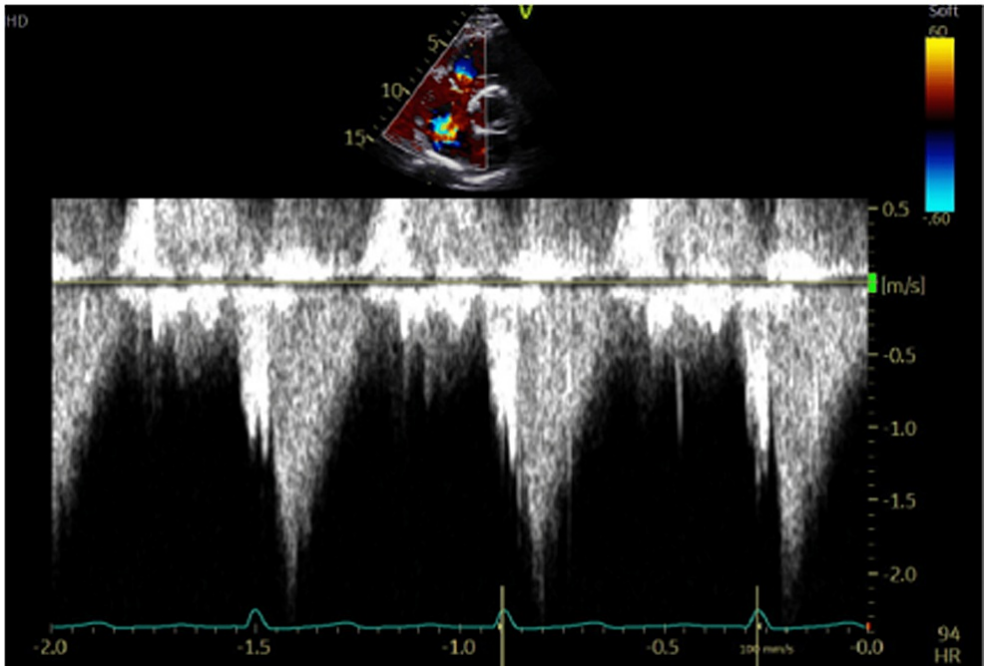
Continuous wave Doppler demonstrating a dense, triangular jet

**Figure 4:**
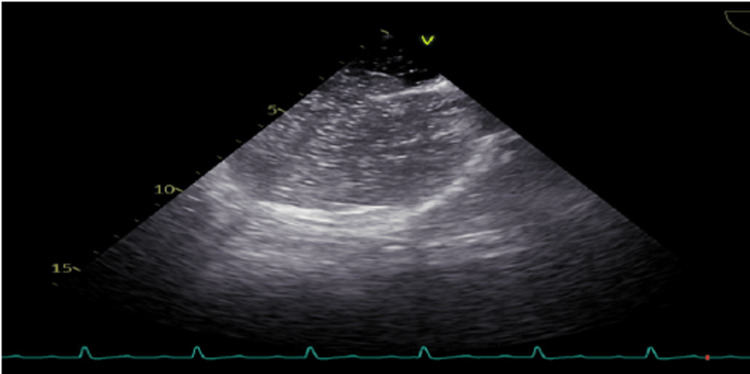
Transesophageal echocardiogram (TEE) demonstrating right-to-left shunt through patent foramen ovale on TEE with bubble study

A transthoracic echocardiogram (TTE) was performed and revealed a flail tricuspid valve (Figure 1). There was also severe regurgitation, as demonstrated by a color Doppler (Figure 2). The left ventricular ejection fraction was maintained at 60-65% with only mild enlargement of the right ventricle and right atrium. Color flow Doppler and pulse Doppler interrogation revealed predominantly right-to-left shunting (Figure 3). A formal transesophageal echocardiogram (TEE) was done and showed findings in the figures below with evidence of patent foramen ovale (PFO) with a tunnel length of 22 millimeters and a diameter of 10 mm (Figure 4). Cardiothoracic surgery was consulted for the repair of the valve as well as the PFO. Given the severe hypoxic state, venovenous extracorporeal membrane oxygenation (ECMO) was initiated and the patient was subsequently transferred to an ECMO facility. Unfortunately, the patient expired before any surgical intervention.

## Discussion

Per the Advanced Trauma Life Support guidelines, a focused assessment with sonography in trauma (FAST) exam should be performed as an initial evaluation of chest wall trauma. It may reveal pathology such as valvular regurgitation or cardiac tamponade. Formal echocardiography should be used to assess the severity as well as the cause of TR. It may also provide information on hemodynamics. As the right ventricle is compliant, symptoms of tricuspid regurgitation may present later as chronic symptoms of right-sided heart failure [6]. More acutely, patients can present with cardiogenic shock to mild dyspnea on exertion. Other indicators of traumatic tricuspid injury include atrial fibrillation and a new right bundle branch block [7].

Medical management of severe tricuspid regurgitation involves treating with loop diuretics for heart failure. Vasodilators may also be used for pulmonary hypertension. Generally, patients with severe TR who will have left-sided cardiac surgery should also undergo TR repair or replacement [8]. Recommendations for isolated tricuspid valve surgery are not well-established due to differences between the American Heart Association (AHA) and American College of Cardiology (ACC) versus the European Society of Cardiology (ESC) guidelines. According to the 2021 guidelines, the ESC recommends tricuspid valve surgery if the patient is symptomatic, has severe isolated primary TR, and is without severe right ventricular dysfunction [9]. In patients with secondary TR, surgery can be considered to reduce symptoms and recurrent hospitalizations if they are symptomatic. AHA and ACC guidelines include a weak recommendation for patients with severe TR with no or minor function if there is progressive right ventricular dilation or systolic dysfunction [8].

Lesions commonly seen involving the tricuspid valve include chordal rupture, papillary muscle rupture, and leaflet rupture [10]. A flail leaflet can be repaired by plication with or without resection, chordae can be replaced, and papillary muscles can be reconstructed [10]. Unfortunately, our patient had a flail tricuspid leaflet with acute TR. With the presence of a PFO, it resulted in a right-to-left shunt with significant hypoxia.

## Conclusions

Severe tricuspid regurgitation may occur due to various etiologies. In a patient with blunt chest trauma, the diagnosis of flail tricuspid regurgitation must be considered. Additionally, echocardiography should be used to further evaluate additional causes such as atrial and ventricular septal defects. Early detection and management of tricuspid regurgitation are vital in improving patient outcomes as well as quality of life.
